# Fame and future of faecal transplantations — developing next-generation therapies with synthetic microbiomes

**DOI:** 10.1111/1751-7915.12047

**Published:** 2013-04-10

**Authors:** Willem M Vos

**Affiliations:** 1 Laboratory of Microbiology, Wageningen UniversityWageningen, the Netherlands; 2 Department of Veterinary Biosciences, Faculty of Veterinary Medicine, University of HelsinkiHelsinki, Finland; 3 Department of Bacteriology and Immunology, Medical Faculty, University of HelsinkiHelsinki, Finland

## Abstract

While practised for over thousand years, there is presently a renaissance in the interest of using of faecal transplantations to modify the intestinal microbiota of patients. This clinical practice consists of delivering large amounts of bowel microbes in various forms into the intestinal tract of the recipient that usually has been cleared previously. The major reason for the popularity of faecal transplantations is their effectiveness in treating a variety of diseases. Hence, there is a need to develop this procedure to the next level. While there are various developments to select, standardize and store the donor microbiota, it is more challenging to understand the intestinal microbial communities and develop ways to deliver these via robust biotechnological processes. The various approaches that have been followed to do so are discussed in this contribution that is also addressing the concept of the minimal microbiome as well as the production of the synthetic communities that can be instrumental in new therapeutic avenues to modify the intestinal microbiota.

Our intestinal tract is colonized since birth by complex communities of microbes that show specific spatiotemporal organizations, differ in composition between individuals, and contribute to health and disease (Zoetendal *et al*., 2006). Considerable progress has been made in recent years to describe the structure and function of the intestinal microbiota that belong to the major phyla of the *Firmicutes*, *Actinobacteria*, *Bacteroidetes*, *Proteobacteria* and *Verrucomicrobia* (Rajilić-Stojanović *et al*., 2007). However, the majority of the over 1000 species-like groups of the human intestine have not yet been cultured (Zoetendal *et al*., 2008). Facilitated by the advances in sequencing technologies, significant attention has been given to culture-independent and high-throughput approaches that generated important baseline information on the intestinal microbiota composition, the description of a reference metagenome of 3.3 Mb, and its structuring into clusters, termed enterotypes (Qin and the MetaHit Consortium, 2010; Arumugam and the MetaHit Consortium, 2011; Huttenhower and the Human Microbiome Project Consortium, 2012).

The application of the high-throughput technologies confirmed earlier observations that adults have a unique and stable microbiota (Zoetendal *et al*., 1998). This has been recently extended by the highly accurate, reproducible and deep analysis of the microbiota of thousands of adults that revealed all of them to have a different composition (J. Salojarvi and W. M. de Vos, unpublished observations using the HITChip, a phylogenetic microarray) (Rajilić-Stojanović *et al*., 2009). As the systematic analysis of the human microbiota is only recently emerging at this large scale, there are not many studies that addressed the long-term dynamics of the intestinal microbiota. A deeply sampled longitudinal study of two subjects for over a year showed the microbial composition in faecal samples to be rather stable in contrast to that in other body parts, such as skin and oral cavity (Caporaso *et al*., 2011). Similarly, considerable stability of the of the faecal communities was observed in healthy subjects that were followed for over 10 years and maintained their characteristic personal microbiome (Rajilić-Stojanović *et al*., 2012). Other longitudinal studies indicated that the microbiota composition is affected by diet, antibiotic use and intestinal transit but possibly also by less well-studied lifestyle factors, such as time zone travelling (Flint *et al*., 2012; Jalanka-Tuovinen *et al*., 2011). An inherent determinant of this dynamics could be formed by bacteriophages that are detected in faecal samples and metagenomes (Qin and the MetaHit Consortium, 2010; Minot *et al*., 2011; Reyes *et al*., 2012). As typical oscillations have not been observed, it is not yet clear how bacteriophages control the intestinal microbiota but in analogy with other systems, it may be speculated that the intestinal microbiota is in the carrier state with virulent bacteriophages contributing to its stability (de Vos, 1989).

The abundance of accurate approaches to monitor the composition and coding capacity of the intestinal microbiota also greatly advanced the analysis of the differences in intestinal microbiota composition of healthy and diseased subjects. Presently, correlations between specific intestinal microbes or microbial patterns have been determined for several dozens of diseases, varying from severe intestinal inflammations to cancer and obesity, as reviewed recently (de Vos and de Vos, 2012). However, causal relations are scarce and hard to establish, as there are many microbiological, medical and ethical issues to deal with. An exception is the use of faecal transplantations of the intestinal microbiota that is receiving increasing popularity in human interventions since this practice not only provides causal relations but also shows considerable efficacy in treating various diseases, supporting the importance of the intestinal microbiota. Remarkably, there is a large discrepancy between the low-key technology used in this treatment and the sophisticated knowledge of the intestinal microbiome. Hence, this caveat is addressed here with specific attention for next-generation therapies at the interface of medical practice and microbial biotechnology.

## Development of faecal transplantation: from human to animals and back

Long before the discovery of microbes, the concept of transplanting faecal material has been practiced, supporting the often-empirical nature of the medical practice. Records in Chinese medicine documenting the transfer of faecal suspensions date for over 1000 years, as reviewed recently (Zhang *et al*., 2012). This include the first description by Ge Hong, a medical doctor during the Djong-ji dynasty (fourth century), who could cure patients suffering from food poisoning or severe diarrhoea by making them consume faecal suspensions from healthy donors. Similarly, during the Ming dynasty (16th century) Li Shizen provided detailed descriptions of faecal therapies for the effective treatment of certain abdominal diseases, as noted recently (Zhang *et al*., 2012). Remarkably, the concept of transferring intestinal samples in veterinary practice was in use around the same time in Europe where the Italian anatomist Fabricius Aquapendente (17th century) described the practice of inoculating rumen fluid presumably into cows that had lost the capacity to ruminate (Borody *et al*., 2004). This procedure is still practiced today when cud, which is the material that is brought up into the mouth by a cow from its first stomach to be chewed again, is used to inoculate young calves via the oral cavity (Pounden and Hibbs, 1950). In a more extreme situation, this inoculation takes place in adult cows with rumen fluid directly obtained via a fistula from a well-producing cow. This process, also known as transfaunation since both microbes and protozoa are transferred, is an effective therapy in acidosis of cows fed high grain levels that is caused by overgrowth of mainly *Streptococcus bovis* (Klieve *et al*., 2003). In the second part of last century, the practice of faecal transplantation was extended to avian species in the so-called Nurmi concept where newly hatched chicks are inoculated by mixtures of chicken faeces (Nurmi and Rantala, 1973). This effectively increased the colonization resistance and protected the chicken from infections, notably from *Salmonella* spp. Presently, this practice is still in place and freeze-dried preparations of faecal microbiota from pathogen-free chicken are manufactured and commercialized (Stavric, 1992; Nakamura *et al*., 2002; Revolledo *et al*., 2009).

Some 50 years ago, human faecal transplantations were again documented in the medical practice by the work of Dr Ben Eiseman (Eiseman *et al*., 1958). Four enterocolitis patients were treated with faecal enemas (delivery via the colon) and a rapid recovery of all of them was observed. With the knowledge of the present time, these patients probably would have been described as suffering from *Clostridium difficile* infection (CDI). Following this report dozens of reports have appeared showing the success of faecal transplantation in recurrent or chronic CDI and these include over 500 cases as reviewed recently (Gough *et al*., 2011; Borody *et al*., 2012). The various avenues used for these and other transplantations, their success in the medical practice and the options for applying microbial biotechnology are discussed below.

## Faecal transplantation practice

**Figure 1:**
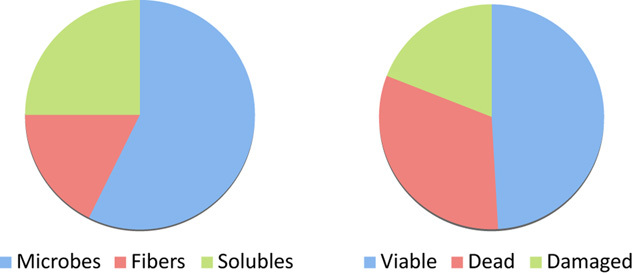
Faecal composition. The distribution of dry weight constituents (left; solubles represent the soluble material) (Stephen and Cummins, 1980) and the differentiation of the microbiota into viable, dead and damaged cells (Ben-Amor *et al*., 2005).

Transplanting faecal microbiota in its simplest form is the consumption of intestinal microbiota. This is a natural process as it is likely to occur in early life when we are born virtually sterile and are colonized rapidly by specific microbial communities that are only found in the human intestine. So we all start our life with at least one, but most likely multiple, faecal transplantations. This is not an issue to be discussed at the first baby visit, dinner table or before breakfast, as was recently noted (Economist, 2012). Hence, there are a great variety of euphemistic descriptions of the material used, including yellow soup, liquid gold or just Julia Flora, termed after the co-worker who donated the original culture (Schoorel *et al*., 1980; P. Heidt, pers. comm.). Similarly, the practice itself enjoys over a dozen of terms, the most recent one being ‘repoopulate’ (Petrof *et al*., 2013). The way the treatment is performed varies considerably and ranges from top to bottom, including simple oral consumption, small intestinal infusion via a nasogastric or naso-duodenal tube, transfer via esophago-gastroduodenoscopy or colonoscopy, or delivery by a colonic retention enema that even can be applied at home. In many cases, the microbial load of the intestinal tract is reduced by a bowel lavage or consumption of laxatives. The delivery mode also explains the wording used for the practice that may vary from faecal duodenal infusion to colonic bowel transplantation. Moreover, often terms as bacteriotherapy or faecal microbiota transplantation are used. However, these do not correctly describe the followed procedure, as the faecal material that is transferred contains more than only microbes as is described below (see also Fig. 1)

## Characterization of faecal material for transplantation

In contrast to the great interest in the microbial make-up, there are only a few data on the abiotic composition of faecal material. In a thorough study of adults consuming a typical British diet (385 g of carbohydrates, 85 g of protein, 108 g of fat and 22 g of dietary fibre) it was found that on average the faecal discharge was close to 100 g per day and consisted for 75% of water. However, when the dry matter was considered, approximately half (55%) consisted of microorganisms, while the remaining part included fibres such as cellulose, as well as soluble material (24%) (Fig. 1A) (Stephen and Cummins, 1980). The latter also included polymers such as mucus, proteins and fat and expectedly all sorts of small soluble molecules including bile acids that constitute a sink for cholesterol degradation products such as catechol, indols and sulfides that generate the familiar odours, and the characteristic short-chain fatty acids that are produced at a level of up to 100 mM in average faecal samples (Flint *et al*., 2012). In most faecal transplantations no efforts are made to purify or enrich the microbial fractions and hence the therapeutic preparations contain all or most of these compounds.

Some pre-treatments are done with the faecal samples that are administrated via the upper intestinal tract and include diluting, blending and filtering the faecal microbiota (Vrieze *et al*., 2012). Protective measures are taken to reduce the exposure to oxygen-rich situations but cannot be prevented. Analysis of the microbiota before or after this treatment did not show major differences (E. G. Zoetendal and W. M. de Vos unpubl. obs.). However, the viability of the preparations has not been tested. This also has not been done for the frozen faecal samples that are used for colonic delivery in CDI patients and also included washing and subsequently storage in cryoprotectants, such as glycerol (Hamilton *et al*., 2012). This is all of relevance, since it has been reported that the majority of faecal microbes are dead or damaged (Fig. 1B). This is not a surprise as the end-of-the-pipeline fermentations that take place in the colon are not there to preserve viability. The solids contain many toxic components, the most relevant is bile that has powerful antimicrobial properties (Begley *et al*., 2005). The viability of faecal microbiota has been determined in Dutch subjects consuming a regular diet using advanced flow cytometry with functional fluorescent probes (Ben-Amor *et al*., 2002). It was observed that about half of the microbes (49%) are dead, one-third are alive (32%), and a smaller fraction is damaged and most likely only can be cultured with specific treatments as described previously (Fig. 1B) (Ben-Amor *et al*., 2002). This is not likely to happen during the present treatments of faecal transplantation that often require manipulations under less strict anaerobic and protecting conditions than the ones used to preserve viability (Ben-Amor *et al*., 2002).

In conclusion, it is fair to assume that the vast majority of the transplanted microbes are dead. It cannot be excluded that these, together with other abiotic compounds present in the faecal samples (Fig. 1A), play a role in the success of the faecal transplantations and this is an area that needs further study. Importantly, the viable microbiota that can be recovered from faecal samples shows a non-uniform distribution with less than expected *Bacteroidetes*, some major *Clostridium* groups and *Bifidobacterium* spp. (Ben-Amor *et al*., 2002). Remarkably, butyrate-producing bacteria such as *Eubacterium hallii* were found to be present in the viable fraction. This is of interest as we recently found that *E. hallii*, which converts lactate and acetate into butyrate, was enriched in the ileal microbiota of metabolic syndrome subjects that were cured from their insulin-resistance by faecal transplantation (Vrieze *et al*., 2012). These differential effects on viability may constitute an important lead for further work that aims to provide cause–effect relations between the transplanted microbiota and the health outcomes.

## Present success of faecal transplantation

Following the success of faecal transplantations in the treatment of colitis and recurrent CDI patients in the last 50 years, a variety of other diseases have been targeted. An important one was the first case report in 1989 by Dr Justin D Bennet of Ulcerative Colitis (UC), a severe colitis of the colon (Bennet and Brinkman, 1989). He suffered himself from UC and after describing many unsuccessful treatments he was finally cured by repeated colonic application of large amounts of faecal material from a healthy donor. Since then, many applications in CDI, UC and others have been pioneered, mostly as case studies (Borody and Khoruts, 2011). Presently, successful faecal transplantations have been described for approximately half a dozen of diseases, some of which with large numbers of treated patients (Table 1). There seems not to be a great difference in the success rate when using oral, duodenal and colonic delivery as is evident from the treatment of recurrent CDI. However, in all cases specific precautions are taken in the selection of the donor, varying in stringency from general health to large lists of criteria that have to be met, such as absence of HIV and other viruses, intestinal complaints, or unsafe sex or use of illicit drugs (Vrieze *et al*., 2013). Apart from CDI and UC, these include irritable bowel syndrome (IBS) that is also characterized by an aberrant microbial composition (Rajilić-Stojanović *et al*., 2011). In addition, indications exist for the successful treatment of chronic fatigue syndrome (Borody *et al*., 2012) and multiple sclerosis (Borody *et al*., 2003) but these are only based on single studies, as has been reviewed recently (Vrieze *et al*., 2013). An increasing number of studies is also addressing the microbiota composition and function in the donor and the recipient before and after transplantation (Table 2). This is an important addition to the learning curve and should be standard practice for new interventions as it will contribute to further developing faecal transplantations. Some of the most relevant findings are summarized below.

**Table 1:** Diseases treated by faecal transplantation

Disease	N	Delivery	Reference
*C. difficile* infection	70	C	Mattila *et al*. (2012)
*C. difficile* infection	16	D	van Nood *et al*. (2013)
Insulin resistance (MetS)	9	D	Vrieze *et al*. (2012)
Ulcerative colitis	6	C	Borody *et al*. (2003)
Irritable bowel syndrome	30	C	Andrews *et al*. (1995)
Chronic fatigue syndrome	60	C	Borody *et al*. (2012)
Multiple sclerosis	4	C	Borody *et al*. (2003)
Metabolic acidosis	1	O	Schoorel *et al*. (1980)
Recolonization after AD	6	O	van der Waaij *et al*. (1977); Heidt *et al*. (1983)

The number (N) of described cases is indicated as are the different modes of delivery (D, duodenal; C, colonic or caecal; O, Oral). Studies with the largest number of patients reported are listed. AD, antibiotic decontamination.

**Table 2:** Lessons learned from transplantations

Patient	Number	Major change	Reference
CDI	1	Increased diversity	Khoruts *et al*. (2010)
		Increase in *Bacteroidetes*	
CDI	6	Increased diversity – not all successful	Shahinas *et al*. (2012)
		Increase in *Bacteroidetes* 0026; *Firmicutes*	
		Decrease in *Proteobacteria*	
CDI	3	Increased diversity – like donor in 2/3	Hamilton *et al*. (2013)
		Increase in *Bacteroidetes* 0026; *Firmicutes*	
		Decrease in *Proteobacteria* 0026; *Actinobacteria*	
CDI	9	Increased diversity – like donors in 9/9	van Nood *et al*. (2013)
		Increase in *Bacteroides*, some *Firmicutes*	
		Decrease in *Proteobacteria*	
MetS	8	Slightly increased diversity	Vrieze *et al*. (2012)
		Increase in some butyrate producers	

In the over 500 cases where faecal transplantations have been practiced on recurrent or chronic CDI patients, an average disease resolution of over 95% has been reported (Borody and Campbell, 2012). However, this number may suffer from a publication bias as many studies are small or represent successful case reports. Several recent studies have approached faecal transplantation in CDI in a systematic way with many patients. In a study at Helsinki University, a total of 70 recurrent CDI patients were treated by colonic delivery, resulting in 66 cured patients (94%) (Mattila *et al*., 2012). In another study in the Amsterdam Medical Centre, a three-arm study was set up where 42 recurrent CDI patients either received vancomycin, vancomycin and a bowel lavage, or all these treatments followed by duodenal infusion of healthy donor faeces (van Nood *et al*., 2013). After an interim evaluation, the study was stopped by the medical–ethical board since the transplanted patients showed much better recovery than those receiving either of the other treatments – in total 13 of the 16 CDI patients recovered immediately after the faecal transplantation and two of the remaining three after a second infusion. This contrasts markedly with the other treatments that cured either four or three out of 13 with vancomycin, or vancomycin and bowel lavage respectively. Interestingly, the several of these that did not respond to the vancomycin-based treatments received an off-protocol faecal transplantation and 15 of them recovered immediately. Altogether, this resulted in a successful treatment of 30 of the 33 CDI patients (91%). In the latter study the microbiota was studied extensively (van Nood *et al*., 2013; S. Fuentes and W. M. de Vos, unpubl. obs.). The recurrent CDI patients were all characterized by a consistent and reproducible very low intestinal microbiota diversity that was immediately corrected to donor levels after the faecal transplantation and this stable and healthy situation was followed up for 6 weeks in multiple patients. This analysis extended previous studies in a single (Khoruts *et al*., 2010) or limited number of subjects with fresh (Shahinas *et al*., 2012) or frozen faeces (Hamilton *et al*., 2012) (Table 2). A dramatic increase in *Bacteroidetes*, some *Firmicutes*, including butyrate-producing ones, as well as a reduction in pathobionts belonging to the *Proteobacteria* were observed (van Nood *et al*., 2013). This all indicated a drastic conversion from a low diversity but highly inflammatory microbiome into that with the characteristics of a healthy microbiota (De Vos and De Vos, 2012). While some individual differences can be observed, the global picture is the same in this and other reported analyses of the microbiota after CDI transplantations (Table 2). This indicates that the nature of the microbiota (fresh or frozen), the delivery mode (duodenal or colonic), or the location, origin or dietary habits of the donors, do not affect the final outcome. It also confirms the earlier observations that the intestinal ecosystem in the CDI patients is so disturbed that the donor microbes rapidly start occupying the available niches resulting in a normally functioning intestinal microbiota (Khoruts *et al*., 2010).

Most faecal transplantations are performed with stool samples from multiple healthy donors, often family members. Occasionally, transplantations are performed with one or a preferred donor. However, in a recent study a total of 32 recurrent CDI patients were treated with an enema of a mixture of intestinal microbes derived from a single healthy middle-aged donor in 1994, which had been re-cultivated under strict anaerobic conditions for over 10 years (Jorup-Rönström *et al*., 2012). A simple peptone-yeast medium was used for this subculturing that included egg yolk as a source of cholesterol. Usually such culturing steps decrease the microbial diversity rapidly but the effectiveness of this yet undefined mixture appeared to persist, as 22 out of the 32 patients (69%) could be cured. Remarkably, the mixture was administered as a 30 ml suspension by a 50 cm rectal catheter without prior laxation, although precautions were taken to keep the enema as long as possible. This contrasts with other administration procedures that use usually over 100 g of faecal material that is inserted after bowel lavage. This, the unusual way of delivery, and the nature of the inoculum may be among the factors explaining the lower than elsewhere reported curing rates.

The vast majority of the described studies do not have a control group, most likely as it is not ethical to withheld the best treatment from the patients as the experience with the CDI transplantation indicates (van Nood *et al*., 2013). Moreover, the selection of a control treatment is far from simple and specifically not in the case of transplantations. However, an elegant solution has been provided in a large study with patients suffering from metabolic syndrome (MetS; Vrieze *et al*., 2012). Here a control group of nine subjects did not receive faecal transplants from lean donors as the treatment group, but were transplanted with their own faecal samples in a so-called autologous transplantation. Only the treatment group receiving the lean donor transplants showed loss of MetS as evidenced by an increased insulin sensitivity (Table 1). As this study was performed in a blinded way, it represents the first double blind, placebo-controlled faecal transplantation study, adding both credibility and power to the observed results. Moreover, a deep microbiota analysis was also performed in this study and showed less pronounced differences than that after transplantation of the recurrent CDI patients. However, small but reproducible differences could be detected in the cured treatment group, which were absent in the autologous group that received their own microbiota (Vrieze *et al*., 2013). These included an increase of butyrate-producing bacteria (*Roseburia intestinalis* in the lower and *E. hallii* in the upper intestinal tract) that was accompanied by a reduced number of *Proteobacteria*, including *Escherichia coli* (Table 2). This suggests a similar situation, though not so prominent, as observed after the treatment of recurrent CDI patients, viz. a reduction of potential inflammatory Gram-negative bacteria by those that are capable of producing butyrate after faecal transplantation with a healthy donor.

An unusual study that has received only little attention involves the so-called Julia Flora. This term relates to the faecal microbiota from a healthy donor that was maintained in germ-free mice and could be easily delivered via the oral route (Van der Waaij *et al*., 1977). This Julia Flora was used to rescue a 3-year-old boy with short bowel syndrome who suffered from metabolic acidosis due to overproduction of d-lactic acid (Schoorel *et al*., 1980). As described above, acidosis is often encountered in cattle due to *S. bovis* overgrowth (Klieve *et al*., 2003). Here this could be caused by similar mechanism as a higher than normal level of Gram-positive bacteria was observed in the boy's stool. Daily oral administration of the Julia Flora for 5 consecutive days was sufficient to cure the boy and he did not observe any negative effects 9 months after discharge from the hospital (Schoorel *et al*., 1980).

In conclusion, the successes of faecal transplantation range from various levels of colonic inflammation to metabolic diseases. This extends the association of these diseases with the intestinal microbiota by providing a clear causal relation with the faecal components (De Vos and De Vos, 2012). The microbiota analysis confirms and extends this, while providing global insight in how the various taxonomic and functional groups of the recipient are replaced by that of the donor. Moreover, detailed analysis of the microbial changes over time will allow the association of specific bacteria with health status, such as the butyrate-producing *E. hallii* in the case of MetS (Table 2). This type of approaches together with further metagenomic, functional and network analyses will provide a wealth of data that may deliver new leads for defining the minimal microbiome and constructing synthetic microbial communities. This is supported by successes with undefined microbial mixtures, maintained in germ-free mice or subculturing in the laboratory, in curing metabolic acidosis or treating recurrent CDI patients (Schoorel *et al*., 1980; Jorup-Rönström *et al*., 2012). Further steps towards the development of synthetic communities are discussed below.

## Towards synthetic communities

Long before the present interest in our intestinal microbiome and bringing faecal transplantations to the next level, various attempts have been reported on the development of synthetic communities. These are presented here together with the most recently developed mixtures (Table 3).

**Table 3:** Undefined cultures and defined consortia used in microbiota transplantations

Host	Number	Composition	Reference
Germ-free children	2	Two *Bifidobacterium* strains, two spore formers (most likely contaminants of an antibiotic)	Dietrich and Fliedner (1973)
C-section babies	6	Human donor microbiota maintained in germ-free mice	Raibaud *et al*. (1975)
AD patients	5	Human donor microbiota maintained in germ-free mice	van der Waaij *et al*. (1977); Heidt *et al*. (1983)
CDI patients	32	Human donor microbiota subcultured for 10 years	Jorup-Rönström *et al*. (2012)
CDI mice	20	*S. warneri, E. hirae, L. reuteri, Anaerostipes* sp. nov., *Bacteroidetes* sp. nov., *Enterorhabdus* sp. nov.	Lawley *et al*. (2012)
CDI patients	6	*E. faecalis, C. innocuum, C. ramosum, B. ovatus, B. vulgatus*, *B. thetaiotamicron, E. coli* (2), *C. bifermentus, P. productus*	Tvede and Rask-Madsen (1989)
CDI patients	2	***A. intestinalis****, B. ovatus, Bif. adolescentis* (2), ***Bif. longum* (2)**, *Bl. producta*, *C. cocleatum*, *Col. aerofaciens, D. longicatena* (2), *E. coli*, *Eub. desmolans*, ***Eub. eligens****, Eub. limosum*, ***Eub. rectale* (4)***, Eub. ventriosum*, ***F. prausnitzii****, Lach. pectinoshiza, L. paracasei, L. casei, Par. distasonis*, *Raoultella* sp., *R. faecalis, R. intestinalis*, ***Rum. torques* (2)**, *Rum. obeum* (2), *S. mitis*	Petrof *et al*. (2013)

AD, antibiotic decontamination. *E*., *Enterococcus*; *C*., *Clostridium*; *B*., *Bacteroides*; *E*., *Escherichia*; *P*., *Propionibacterium*; *A*., *Anaerostipes*; *Bif*., *Bifidobacterium*; *Col*., *Colinsella*; *Eub*., *Eubacterium*; *F*. *Faecalibacterium*; *Lach*., *Lachnospira*; *Par*., *Parabacteroides*; *R*., *Roseburia*; *Rum*., *Ruminococcus*; *S*., *Streptococcus*; *L*., *Lactobacillus*. The species indicated in bold are the main constituents of the mixtures.

An unusual set of transplantations that followed intensive treatments with antibiotics to decontaminate the intestinal tract, have described as early as 1973. At this time, isolators were developed to protect patients with immunological deficiencies, such as those treated with acute leukaemia. Some of these studies are detailed here, since it seems that these have escaped the attention of those active in the field of faecal transplantations. In an extensive report describing a complete containment system, it was reported that immune-compromised children and adults could enter a germ-free state after long-term antibiotic treatments (Dietrich and Fliedner, 1973). After the appropriate therapy and cure of the disease in these patients, they had to be colonized in order to enter the ordinary environment. This was performed by a cascade of oral inoculations of single bacterial strains (*Bifidobacteria*, *Lactobacilli*, *E. coli* and *Enterococci*), followed by a rectal insertion of a faecal sample from a healthy subject. It was found that each bacterium colonized immediately without symptoms of disease in two of such germ-free children. A single case was described in more detail and included a gnotobiotic child that had been on oral treatment of 500 mg day^−1^ of gentamicin, which was probably contaminated by two aerobic spore-forming bacilli that subsequently colonized. This child received first 10^6^ and subsequently 10^10^ cells of different strains of *Bifidobacteria*, delivered with a milk diet. These inoculated strains grew out to up to approximately 10^8^–10^10^ bifidobacterial cells per gram faecal material, outcompeting the spore formers by a factor of 10–1000. After 4 months the faecal transplantation was performed and the child left the isolator (Dietrich and Fliedner, 1973).

Around the same time, faecal transplantations were used to conventionalize caesarean-section (C-section) delivered children, suspected of congenital combined immunodeficiency (Raibaud *et al*., 1975). Ten days after their delivery, these were fed a suspension of intestinal content of germ-free mice that had been colonized by a diluted human faecal sample. This is the first description of the oral transplantation by a humanized microbiota derived from mice. A similar approach was followed a few years later when a humanized and non-pathogenic microbiota was obtained from a healthy donor, named J.F. (Van der Waaij *et al*., 1977). The generated microbiota was termed first J.F. flora, and later named Julia Flora (see above and Table 2) and also Human Donor Flora (HDF). Remarkably, during the maintenance in mice, *E. coli* was lost as a major component of the microbiota and mainly consisted of anaerobic bacteria (Heidt *et al*., 1983). We now know from various studies that the human microbiota in germ-free animals may change rapidly in structure and function (El Aidy *et al*., 2012). This J.F. flora was used as an oral inoculum to repeatedly (up to five times) recolonize a series of five immunocompromised and hence isolator-kept patients that had been receiving antibiotics in a treatment known as antibiotic decontamination (AD). While virtually sterile before transplantation, the colonic counts rapidly increased after the colonization. However, it was noted that the colonization resistance of the transplanted patients was not as good as that of a normal healthy subject (Van der Waaij *et al*., 1977). Whether this is due to the composition of the Julia Flora or the impact of the compromised hosts cannot be said but it appeared that the quality of the J.F. flora (then termed Julia Flora) was sufficiently high to cure metabolic acidosis in a young boy (Schoorel *et al*., 1980; see above).

A series of early but very important experiments with synthetic communities were reported around the same time by Tvede and Rask-Madsen (1989). From 20 intestinal single strain isolates, they selected 10 strains, several of which showed inhibitory activity against *C. difficile* isolated from the six patients with chronic CDI. These patients were treated successfully with faecal samples from healthy relatives or the strain mixture that were all rectally installed. One patient was treated with a faecal enema only, one with a faecal enemas that were unsuccessful and subsequently with the strain mixture, and the remaining four only with the strain mixture that was rectally installed (Table 3). Faecal samples of the patients before these treatments did not contain detectable *Bacteroides* spp. in line with later observations described above (Table 2). This is the first description of a synthetic mixture of intestinal bacteria that shows success in curing five CDI patients from their recurrent CDI. It also showed that the developed mixture was superior to faecal transplantation in the one case tested. The mixture consisted of three *Bacteroides* spp. that all were inhibited by the patients' *C. difficile*. However, one *E. coli* strain as well as the *Clostridium bifermentans* and *Peptostreptococcus productus* strains showed antagonistic activity against *C. difficile*. In this context it is relevant to note that recently a two-component modified antimicrobial peptide, thuricin CD, was detected in an intestinal *Bacillus thuringiensis*, which showed strong inhibitory activity against *C. difficile in vitro* and in a distal colon model (Rea *et al*., 2010; 2011). A long-term follow-up of the patients treated with the synthetic mixture was described and included analysis of the faecal samples by anaerobic culturing (Tvede and Rask-Madsen, 1989). It was observed that mainly the *Bacteroides* spp. colonized and it was suggested that colonization by these bacteria may provide a natural defence against *C. difficile* although synergistics effect of the inhibiting species could not be excluded. These hallmark findings attracted quite some attention but also were criticized as is usually the case when novel approaches are reported. The suggestions that oral therapies with probiotic bacteria or yeasts could be a better approach were adequately rebutted (Seal *et al*., 1989; Tvede and Rask-Madsen, 1989). The question whether 10 strains are too many or too few has not yet been answered, even not with the molecular approaches available now.

As may be expected from the abundance of the recurrent of chronic CDI, most recent studies in developing synthetic communities have been focusing on this disease. An elegant mouse study was recently reported and included a CDI model that was tested with the best synthetic communities selected via some combinatorial approach. Best results were obtained with a mixture of six species that included some new mouse isolates (Lawley *et al*., 2012). How the mouse isolates will perform in the human intestinal tract is not known. Similarly, it remains to be seen what regulatory hurdles have to be overcome before human trials can be done with this mixture of mouse strains. However, this study clearly demonstrates the success of an avenue that can be followed to develop, maintain and test synthetic communities for treating CDI patients. This extends the other recent study where a human intestinal microbiota was maintained for over 10 years in the laboratory (Jorup-Rönström *et al*., 2012; see above). A recent human pilot study re-addressed the issue of using synthetic microbial communities for curing CDI (Petrof *et al*., 2013). A total of 33 strains of a variety of bacterial groups were isolated from the stool of a healthy donor, combined and cultured, and subsequently used to treat two patients with recurrent CDI who had failed to respond to antibiotic treatments (Table 3). Following delivery by colonoscopy, it was found that the patients were cured while analysis of the resulting intestinal microbiota indicated establishment of some of the new strains. This proof-of-principle study confirms the earlier results of Tvede and Rask-Madsen (1989).

## Future of faecal transplantations

Experimental observations ranging from traditional Chinese medicine to those of modern evidence-based medicine, have provided clear evidence that faecal transplantation may work in a series of diseases, recurrent or chronic CDI being the best investigated one (Kelly, 2013; van Nood *et al*., 2013). The early results initiated in the late seventies and early eighties of large century, with undefined mixtures as well as defined consortia as pioneered by Tvede and Rask-Madsen (1989), indicate strongly that synthetic communities are a feasible approach. This is confirmed by the recent but still anecdotal studies in mice and man (Table 3). There is a clear need to further develop the concept of synthetic microbial communities to treat CDI and possibly other diseases.

The question on how many strains or species are needed, raised over a dozen years ago is still actual. This brings forward the concept of the minimal microbiome that can be defined as the smallest set of microbes and/or microbial functions needed to develop a stable community. Large data sets of microbial communities, their metagenomes and function are being collected worldwide. These can be mined for the networks of microbes and their functions that may provide leads on how such minimal microbiome would look like. Similarly, the analysis of the interactions between intestinal microbes and the host are being studied at a systems level (Martins dos Santos *et al*., 2010). This will allow the identification of microbial components that can be used to predict the minimal microbiomes. Moreover, there is a great variety of new avenues have been developed that can be followed to isolate and culture new microbial strains, varying from germ-mice to functionalized solid surfaces (Ingham *et al*., 2007; 2012; Goodman *et al*., 2011). Hence, there are plentiful possibilities to develop these minimal microbiomes into products based on synthetic microbial communities.

The advantages of synthetic microbial communities are clear as the composition of the synthetic mixtures can be controlled, tested extensively for the absence of undesired pathogens and viruses, and can be reproducibly manufactured. Moreover, the viability can be controlled and optimized. A large number of industrial fermentations rely on this principle and include the aseptic production of freeze-dried or frozen combinations of viable strains such as in the production of multiple strain starter cultures or probiotics (de Vos, 2011). Implementation of these synthetic microbial communities in next-generation therapies would greatly benefit the patients, further advance our understanding of the intestinal microbiome, and intensify the relation between medical practice and microbial biotechnology.
